# NanoSIMS imaging of lipid absorption by intestinal enterocytes

**DOI:** 10.1016/j.jlr.2022.100290

**Published:** 2022-09-28

**Authors:** Kai Chen, Wenxin Song, Robert Russell, Alessandra Ferrari, Tamim Darwish, Peter Tontonoz, Stephen G. Young, Haibo Jiang

**Affiliations:** 1 Department of Chemistry, The University of Hong Kong, Hong Kong, China; 2 School of Molecular Sciences, The University of Western Australia, Crawley, Australia; 3 David Geffen School of Medicine, University of California, Los Angeles, CA, USA; 4 National Deuteration Facility, ANSTO, Lucas Heights, Australia

**Figure:**
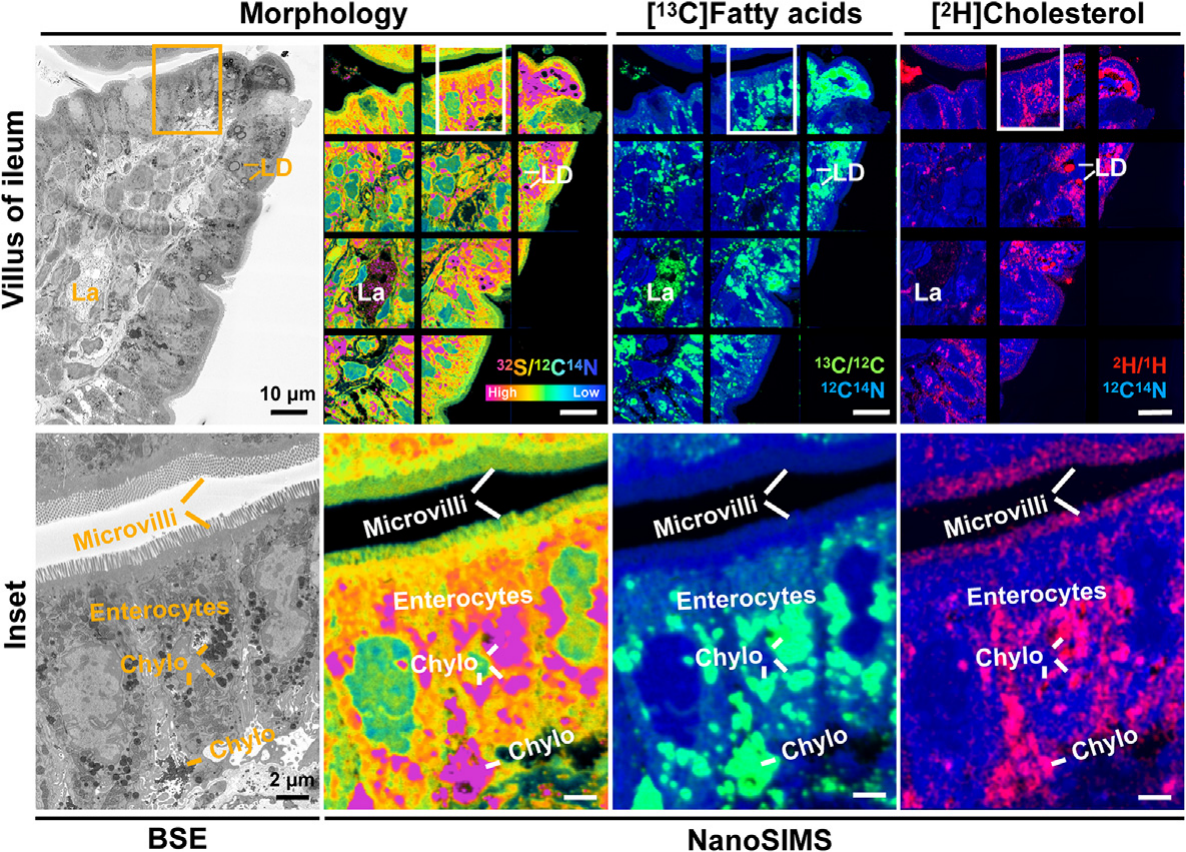

Absorption of lipids by intestinal enterocytes is crucial for growth and development and is highly relevant to human metabolic diseases. Hence, the molecules and mechanisms for lipid absorption have been studied intensively. Electron microscopy has been useful for exploring intestinal lipid absorption (1), but high-resolution images of the absorption of specific lipids have been lacking.

We have studied the uptake of stable isotope-labeled lipids by intestinal enterocytes with correlative backscattered electron (BSE) and nanoscale secondary ion mass spectrometry (NanoSIMS) imaging. BSE images of sections of intestine provide ultrastructural morphology. NanoSIMS images of the same section show the elemental and isotopic content of the sections (e.g., ^1^H^–^, ^2^H^–^, ^12^C^–^, ^13^C^–^, ^12^C^14^N^–^, ^32^S^–^) at ∼50-nm resolution, making it possible to define both enterocyte morphology and the location of stable isotope-labeled lipids within enterocytes (2). This correlative imaging methodology makes it possible to match high-resolution chemical information from NanoSIMS analyses with the ultrastructural detail of BSE images.

In the current study, a wild-type mouse was given (by gastric gavage) 20 mg of [^13^C]-mixed fatty acids and 10 mg of [^2^H]cholesterol. After 3 h, the mouse was euthanized, and 500-nm-thick resin-embedded sections of the ileum were prepared for BSE/NanoSIMS imaging (3). In the upper row, BSE and NanoSIMS images show a villus (stained with thiocarbohydrazide and osmium tetroxide). The boxed area in the upper row is shown at higher magnification in the lower row. BSE images show the ultrastructure features of enterocytes, cells of the lamina propria, and a lacteal-containing chylomicrons (Chylo). Enterocytes had apical microvilli, electron-dense lipid droplets (LDs), and Chylo. A ^32^S/^12^C^14^N NanoSIMS image was useful for villus morphology. LDs and Chylo had a high ^32^S/^12^C^14^N ratio (reflecting ^32^S enrichment from thiocarbohydrazide). ^13^C/^12^C ratio images and ^2^H/^1^H images (superimposed on ^12^C^14^N images) show distributions of [^13^C]fatty acids and [^2^H]cholesterol. Both lipids were enriched in cytosolic LDs and Chylo, but there were differences in the patterns of ^13^C and ^2^H enrichment. For example, [^2^H]cholesterol was preferentially enriched in the microvilli. Our correlative BSE/NanoSIMS approach will be very useful for investigating the impact of genetic interventions and drug therapy on lipid absorption in the intestine.

**EQUIPMENT:** NanoSIMS 50L (CAMECA), Verios XHR SEM (FEI).

**REAGENTS:** [^13^C]Mixed fatty acids (Cambridge Isotope Laboratories), [^2^H]cholesterol (uniformly labeled) was prepared by the ANSTO’s National Deuteration Facility, cofunded by the National Collaborative Research Infrastructure Strategy.

## Conflict of interest

The authors declare that they have no conflicts of interest with the contents of the article.
